# Mono-pharmacy

**Published:** 1890-10

**Authors:** 

**Affiliations:** Brussels


### MONO-PHARMACY.

Dr. Gailliard, Brussels.

(Translated from the Spanish by E. A. P.)

One of the Hahnemannian reforms the influence of which has been felt in all medical schools, and later most universally accepted is mono-pharmacy.

It is a principle most necessary to be maintained with great integrity, not for respect which is due to our master nor for tradition's sake, but because it is absolutely rational, being entirely favorable to homœopathic therapeutics.

Mono-pharmacy *means* not the administration of one simple remedy for the cure of a disease, but the successive administration of remedies always *simple*, prescribed according to indications as they are presented.

Administration at the same time and the alternation of several remedies, as recommended by some practitioners, constitute a most irrational proceeding that brings us again to the quackery which reigned during the beginning of the century.

All that can be said of complex medicines is that they represent an odious attempt to return to poly-pharmacy so condemned by Hahnemann.

---

*Niccolum* in right-sided sore throats, when the affected side is very sensitive to touch, externally.

---

For odontalgia depending upon caries of teeth; pain shoots from teeth to ear. Kreosote.

---

During dentition child is fretful and irritable and sleepless, Chamomilla failed, Kreosote cured.

